# Short-term locomotor adaptation to a robotic ankle exoskeleton does not alter soleus Hoffmann reflex amplitude

**DOI:** 10.1186/1743-0003-7-33

**Published:** 2010-07-26

**Authors:** Pei-Chun Kao, Cara L Lewis, Daniel P Ferris

**Affiliations:** 1 School of Kinesiology, University of Michigan, Ann Arbor, Michigan 48109-2214, USA; 2 College of Health & Rehabilitation Sciences: Sargent College, Boston University, Boston, Massachusetts 02215, USA

## Abstract

**Background:**

To improve design of robotic lower limb exoskeletons for gait rehabilitation, it is critical to identify neural mechanisms that govern locomotor adaptation to robotic assistance. Previously, we demonstrated soleus muscle recruitment decreased by ~35% when walking with a pneumatically-powered ankle exoskeleton providing plantar flexor torque under soleus proportional myoelectric control. Since a substantial portion of soleus activation during walking results from the stretch reflex, increased reflex inhibition is one potential mechanism for reducing soleus recruitment when walking with exoskeleton assistance. This is clinically relevant because many neurologically impaired populations have hyperactive stretch reflexes and training to reduce the reflexes could lead to substantial improvements in their motor ability. The purpose of this study was to quantify soleus Hoffmann (H-) reflex responses during powered versus unpowered walking.

**Methods:**

We tested soleus H-reflex responses in neurologically intact subjects (n=8) that had trained walking with the soleus controlled robotic ankle exoskeleton. Soleus H-reflex was tested at the mid and late stance while subjects walked with the exoskeleton on the treadmill at 1.25 m/s, first without power (first unpowered), then with power (powered), and finally without power again (second unpowered). We also collected joint kinematics and electromyography.

**Results:**

When the robotic plantar flexor torque was provided, subjects walked with lower soleus electromyographic (EMG) activation (27-48%) and had concomitant reductions in H-reflex amplitude (12-24%) compared to the first unpowered condition. The H-reflex amplitude in proportion to the background soleus EMG during powered walking was not significantly different from the two unpowered conditions.

**Conclusion:**

These findings suggest that the nervous system does not inhibit the soleus H-reflex in response to short-term adaption to exoskeleton assistance. Future studies should determine if the findings also apply to long-term adaption to the exoskeleton.

## Background

Many research groups are developing robotic lower limb exoskeletons to assist in locomotion training after neurological injury [1-6]. The exoskeletons are intended to reduce manual effort from therapists and improve rehabilitation outcomes. Though reducing manual effort from therapists is clearly being achieved by current devices, results for improving rehabilitation outcomes are still equivocal. Studies have demonstrated that the choice of computer control algorithms for robotic gait devices can affect the process of motor learning to robotic assistance [2,7-11]. However, there is no clear theory on how different control algorithms specifically alter mechanisms or aspects of neural control [12,13]. To design better robotic gait devices that can enhance therapy, it is critical to identify neural mechanisms that govern locomotor adaptation to robotic assistance.

In recent studies from our laboratory, we examined how healthy young subjects adapted to a robotic ankle exoskeleton during walking [14,15]. The exoskeleton provided plantar flexor torque under proportional myoelectric control of soleus electromyographic (EMG) activation. We have focused on the ankle joint because it produces a majority of the positive mechanical work during stance in human walking [16] and insufficient plantar flexor torque generation has been shown to be a major factor limiting mobility after neurological injuries [17-19]. When the robotic assistance was first introduced, subjects walked on the ball of their foot during stance due to the increased plantar flexion torque. After two thirty-minute training sessions three days apart, subjects had reduced soleus muscle activation by ~35% and walked smoothly with the exoskeleton mechanical assistance. A large portion of soleus muscle activation is a direct result of proprioceptive feedback, including the stretch reflex response [20-27]. Thus, the nervous system could inhibit reflex activation during walking with the exoskeleton as a mechanism for reducing soleus recruitment.

Increased stretch reflex inhibition with robotic exoskeleton training would be particularly relevant to gait rehabilitation for individuals after neurological injuries. Individuals who had stroke, spinal cord injury, cerebral palsy, and traumatic brain injury often demonstrate abnormally high stretch reflexes that substantially affect their movement capabilities [28-34]. A number of research groups have been investigating training methods to inhibit reflexes and their results demonstrated that reflex responses can be manipulated both in patient populations [28,35-37] and neurologically intact subjects [38-42]. Chen et al (2006) concluded that conditioning of reflex responses in a rat model can improve functional locomotion after spinal cord injury [37]. If a robotic exoskeleton could be used to induce an alteration of reflex responses during human walking, it would have considerable potential as an aid for gait rehabilitation in addition to reducing manual assistance from the therapists. The added mechanical torque provided by the robotic exoskeleton may enhance motor adaptation as subjects would need to tune their muscle activations correctly by normalizing the exaggerated reflexes.

The purpose of this study was to quantify soleus reflex responses in neurologically intact subjects trained to walk with the robotic ankle exoskeleton. By identifying how devices modify musculoskeletal and neural systems with use in neurologically intact subjects, researchers and clinicians have a much better chance of determining which patient populations might benefit from practice with the robotic devices. We used the Hoffmann (H-) reflex, an electrical analogue of the stretch reflex, to examine soleus reflex responses during walking both with the exoskeleton powered and with the exoskeleton unpowered. The H-reflex is elicited by stimulating the afferent nerve (Ia sensory) directly and bypassing the muscle spindle. H-reflex measurements have been extensively used to study how the stretch reflex is modulated centrally [43-45]. The H-reflex is highly task-dependent and is modulated frequently both within a gait cycle and during different motor behaviors [43,44,46-49]. A reduction in H-reflex amplitude has been associated with mastering new motor tasks such as balancing during standing [39,40], perturbed cycling [38], and backward walking tasks [41,50]. In a pilot study, a single subject that had trained with the ankle exoskeleton for several years demonstrated a much lower H-reflex amplitude in proportion to the background EMG during powered walking compared to during unpowered walking [51]. Based on that finding, we hypothesized that subjects would have lower H-reflex magnitudes when normalized to background soleus activity during adapted powered walking than during unpowered walking. In this study, we tested eight subjects who had trained to walk with the robotic ankle exoskeleton for two training sessions. A previous study demonstrated that healthy subjects reached steady-state dynamics of powered walking within the two thirty-minute training sessions [14]. This adaptation period might be enough to elicit a change neurologically because further biomechanical modifications would be relatively small and/or require much longer training periods.

## Methods

### Subjects

Eight healthy, neurologically intact subjects (4 male, 4 female, age 23.6 ± 7.3 years, height 174.2 ± 11.4 cm, mass 70.6 ± 15.3 kg, mean ± SD) gave written informed consent and participated in the study. The University of Michigan Medical School Institutional Review Board approved the protocol, and the study conformed to the standards set by the Declaration of Helsinki.

### Experimental design and protocol

**Figure 1:**
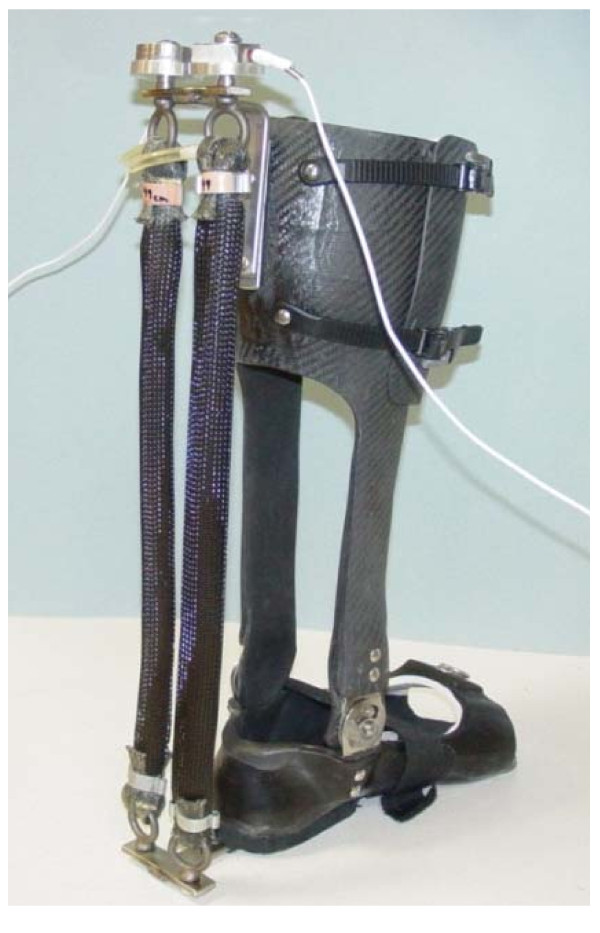
**Subjects wore a custom fit orthosis on their left lower limb**. The orthosis was hinged at the ankle to allow free sagittal plane rotation. Soleus EMG activation was recorded and processed to be used to control air pressure in the artificial pneumatic muscles proportionally. As air pressure increased, the artificial muscles started to develop tension and become shortened, allowing the powered exoskeleton to provide plantar flexor torque controlled by soleus muscle activation.

We constructed a custom-made orthosis (Figure 1) for the left lower limb of each subject. The exoskeleton consisted of a carbon fiber shank section and a polypropylene foot section. A metal hinge between the sections allowed free sagittal plane rotation of the ankle joint. Two artificial pneumatic muscles attached to the exoskeleton provided substantial plantar flexor torque. During powered walking, the peak plantar flexor torque provided by the ankle exoskeleton was ~47% of the total ankle joint moment at push-off [15]. Details of the design and performance of the exoskeleton are documented elsewhere [52-54]. We implemented proportional myoelectric control (i.e., amplitude and timing) of the artificial muscles through desktop computer and real-time control board (dSPACE Inc.). A custom real-time computer controller regulated air pressure in the artificial plantar flexor muscles proportional to the processed soleus electromyographic signals (EMG) via a pressure regulator. The EMG signal from the soleus was high-pass filtered with a second-order Butterworth filter (20-Hz cutoff frequency) to remove movement artifact, full wave rectified, and low-pass filtered with a second-order Butterworth filter (10-Hz cutoff frequency) to smooth the signal. Adjustable gains scaled the control signals and a threshold cutoff eliminated background noise.

Soleus H-reflex was tested while subjects walked with the exoskeleton on the treadmill at 1.25 m/s, first without power (first unpowered), then with power (powered), and finally without power again (second unpowered). Before the testing of soleus H-reflex, subjects had completed two 30-minute treadmill training sessions for walking with the powered ankle exoskeleton controlled by soleus EMG [14,15]. In addition, on the day of soleus H-reflex testing, subjects were given time (i.e., 5 minutes for unpowered conditions and 15 minutes for the powered condition) to re-familiarize themselves to walk with the exoskeleton prior to the nerve stimulations. The same protocol of soleus H-reflex testing repeated in the second unpowered condition was for monitoring the influence of multiple stimuli on the H-reflex amplitudes (e.g., homosynaptic depression) [55].

### Data acquisition and analysis

We collected ankle kinematics, artificial muscle force, electromyography (EMG) and ground reaction forces while subjects walked on a custom-constructed force-measuring split-belt treadmill. The three-dimensional kinematic data were collected by using 8-camera video system (120 Hz, Motion Analysis Corporation, Santa Rosa, CA). Artificial muscle force data were collected with force transducers (1200 Hz, Omega Engineering) mounted on the bracket of orthosis. We placed bipolar surface electrodes on the left shank to record EMGs (1200 Hz, Konigsberg Instruments Inc.) from tibialis anterior (TA), soleus (SOL), medial gastrocnemius (MG), lateral gastrocnemius (LG).

#### Soleus H-reflex measurements

**Figure 2:**
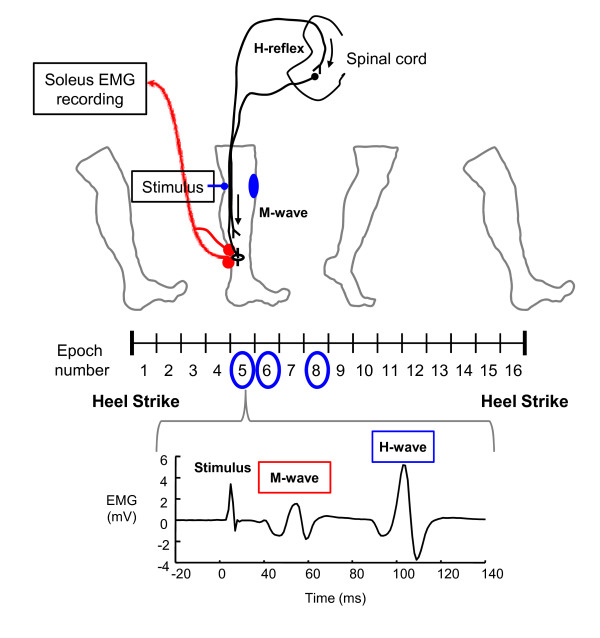
**Soleus H-reflexes were evoked at epoch 5, 6, and 8 (circled)**. We stimulated the tibial nerve with a cathode placed in the popliteal fossa and an anode on the patella. The effective stimulus intensity used for the H-reflex measurements was the intensity to evoke a corresponding M-wave that is 25% of *M*_*max *_for that epoch. We only accepted the measurements of H-waves where their preceding M-waves were 25 ± 10% of the corresponding M_*max*_.

We elicited the soleus H-reflex by stimulating (DS7AH constant current stimulator, Digitimer Ltd.) the tibial nerve with a cathode placed in the popliteal fossa and an anode (7-cm diameter) on the patella (Figure 2). The electrical stimulus was a 1-millisecond monophasic square pulse. We located the optimal site of tibial nerve stimulation using the criterion that a larger M-wave amplitude could be elicited at the same low intensity of stimulus. Before the walking trials, we measured the peak-to-peak amplitudes of M and H waves from surface electrodes (2000 Hz) across different stimulation intensities to gather a standing H-reflex and M-wave recruitment curve.

For the walking trials, we tested the soleus H-reflex in the 3 conditions (first unpowered, powered and second unpowered). We used a footswitch (B&L engineering) to detect heel strikes in real time and estimated the duration of a gait cycle from at least 90 strides in each condition. We divided the gait cycle into 16 equal epochs (10 epochs in the stance). The majority of powered assistance occurred at the middle to late stance, and this was the time period of the largest reductions in the soleus muscle activation [14,15]. Because a large number of stimuli can inhibit H-reflex responses and be uncomfortable for subjects, we evoked soleus H-reflexes for only three epochs: two during mid-stance (epoch 5 and 6) and one during late stance (epoch 8).We used a custom-written program and a real-time control board (dSPACE Inc.) to control the timing of electrical stimuli and to measure the resulting M-wave and H-wave peak-to-peak amplitudes (2000 Hz). We randomly dispersed the stimuli to each of the 3 epochs. The program sent a stimulus at least every 4 seconds.

The size of the M-wave as a percentage of the maximal M-wave (i.e., *M*_*max*_, maximal evoked muscle response) has been used regularly to control constant effective stimulus intensity to the afferent nerve [43,47,49,56]. While walking, the relative movement between stimulating electrode and the nerve may change *M*_*max *_over a stride [49]. To account for changes in *M*_*max*_, we first collected *M*_*max *_data (3 *M*_*max *_measurements) of each epoch by delivering a larger stimulus than the one evoked *M*_*max *_during quiet standing (at least 1.2 times of stimulation intensity for evoking *M*_*max *_during quiet standing).

The effective stimulus intensity used for the H-reflex measurements was the intensity to evoke a corresponding M-wave that is 25% of *M*_*max *_for that epoch. The program monitored the peak-to-peak amplitude of the M-wave produced by the stimulus, and calculated the ratio of the M-wave amplitude to the *M*_*max *_of that epoch. We only accepted H-reflex measurements where the M-wave was 25 ± 10% of the corresponding *M*_*max*_. To ensure constant stimulus intensity over the gait cycle, we manually adjusted the intensity of subsequent stimuli if the ratio was not within the range of 25 ± 10%. We collected 10 measurements of H-reflex where the corresponding M-wave was 25 ± 10% of *M*_*max *_in each epoch.

For background soleus EMG amplitudes, we calculated the mean of rectified averaged soleus EMG of each time epoch. We normalized the H-reflex amplitudes and mean EMG measurements to the *M*_*max *_for that time epoch. This procedure corrected for changes in H-reflex and background EMG values due to movement of the muscle fibers relative to the recording electrodes [49]. Since the H-reflex amplitude depends on the background level of motor activity [56], we calculated the ratio of H-reflex amplitude to its corresponding background EMG amplitude. Thus, the variables we derived were H-wave amplitude (H/M_max_), background EMG amplitude (EMG/M_max_), and the ratio of H-wave and background EMG (H/EMG). To reduce the inter-subject variability, we then normalized the H-reflex, mean EMG amplitudes and the ratio between H-reflex and background EMG in each condition to the values of the first unpowered condition.

### Statistics

We performed Friedman tests to test for differences in normalized H-reflex amplitudes, soleus EMG amplitudes and the ratio between H-reflex and background EMG at the three epochs among the three conditions (first unpowered, powered, and second unpowered). For the small sample size, we chose the nonparametric methods because the validity of this approach does not depend crucially on normality assumption. We set the significance level at *p *< 0.05. If a main effect (i.e., condition) was detected, we used Wilcoxon signed ranks tests to discriminate differences between the powered condition and each of the two unpowered conditions (i.e., powered vs. first unpowered, powered vs. second unpowered) with Bonferroni's correction (adjusted α = 0.025). All statistical analyses were performed in SPSS statistics version 17.0 (SPSS Inc., Chicago, Illinois).

## Results

**Figure 3:**
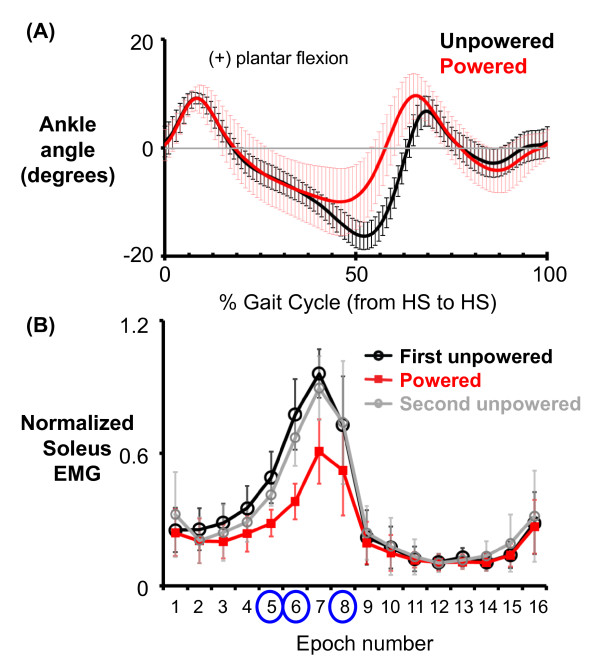
**Ankle joint angle profile (A) and normalized soleus EMG (B)**. Data are the average of all subjects. (A) Ankle joint angle profiles are shown for unpowered (black) and powered condition (red). The error bars represent ± 1 standard deviation. Positive values indicate ankle plantar flexion. (B) Normalized soleus EMG of each time epoch was shown for the first unpowered (black), powered (red), and second unpowered (grey). Epoch 5, 6, and 8 (circled) were the points in time when we performed the H-reflex measurements.

**Figure 4:**
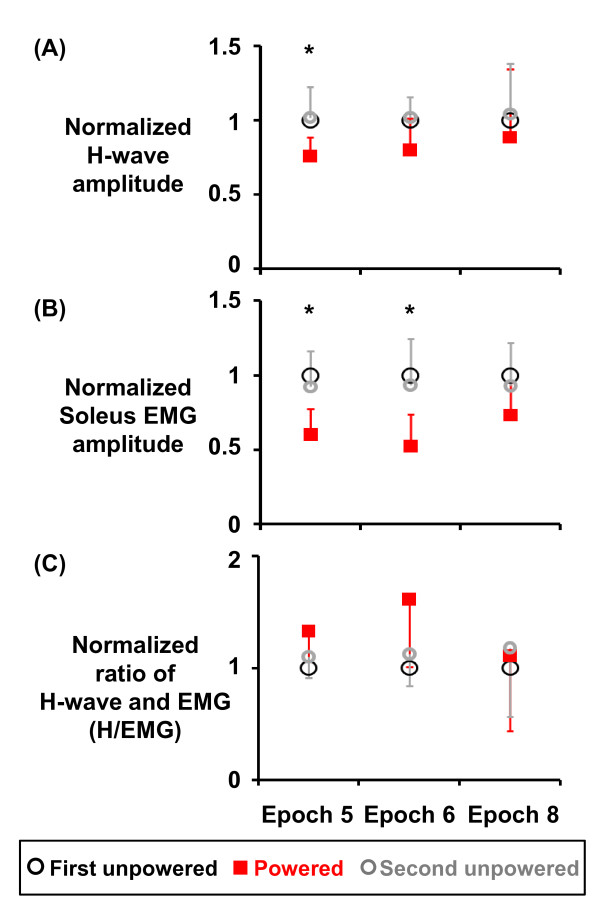
**Normalized H-wave amplitude (A), normalized soleus EMG amplitude (B), and normalized ratio of H-wave amplitude to background EMG (C)**. Amplitudes of H-wave and soleus rectified EMG were first normalized to the peak-to-peak amplitude of *M*_*max *_of that time epoch. To reduce the inter-subject variability, we then normalized the amplitudes in each condition to the values of the first unpowered condition. Thus, the normalized data in the first unpowered condition were 1.0 (100%) for the three epochs.

When the robotic plantar flexor torque was provided, subjects walked with decreased soleus EMG and different ankle joint kinematics at late stance (Figure 3). Compared to the unpowered condition, subjects had similar ankle joint angle profiles during initial to middle stance but the ankle angle profiles deviated from the unpowered ankle angle profiles at epoch 7 (Figure 3A). In addition, the soleus activation was significantly lower in the powered condition for epochs 5 (0.60 ± 0.17; Friedman test, *p *= 0.002; both Wilcoxon signed ranks tests, *p *< 0.025), epoch 6 (0.52 ± 0.21; Friedman test, *p *= 0.002; both Wilcoxon signed ranks tests, *p *< 0.025) and epoch 7 (0.65 ± 0.22; Friedman test, *p *= 0.018; both Wilcoxon signed ranks tests, *p *< 0.025) but not for epoch 8 (0.73 ± 0.22, Friedman test, *p *= 0.18) and the rest of the epochs in stance compared to the two unpowered conditions (Figure 3B, Figure 4B). The soleus EMG amplitudes as well as H-wave amplitudes in the first unpowered condition were equal to 1.0 (100%) for the three epochs because we normalized the data in each condition to the first unpowered condition.

The reduction in soleus EMG activation was much more than the reduction in H-wave amplitude during powered walking. Subjects had significantly lower H-wave amplitudes at epoch 5 (0.76 ± 0.13; Friedman test, *p *= 0.021; both Wilcoxon signed ranks tests, *p *< 0.025) but not at epoch 6 (0.80 ± 0.22, Friedman test, *p *= 0.066) and epoch 8 (0.88 ± 0.46, Friedman test, *p *= 0.867) during powered walking (Figure 4A). Compared to the 27-48% of decrease in soleus EMG activation, H-wave amplitudes were only lowered by 12-24% in the powered condition. Thus, the ratio of H-wave amplitude and background soleus EMG amplitude during powered walking (epoch 5: 1.33 ± 0.26, epoch 6: 1.62 ± 0.60, epoch 8: 1.11 ± 0.67) were not significantly different from the two unpowered conditions (Figure 4C). A condition effect was detected in the epoch 5 (Friedman test, *p *= 0.028) but not in the epoch 6 (Friedman test, *p *= 0.066) and epoch 8 (Friedman test, *p *= 0.651). For further comparisons at epoch 5, the ratio of H-wave and soleus EMG in the powered condition was significantly different from the ratio in the first unpowered condition (Wilcoxon signed ranks test, *p *= 0.012) but not the second unpowered condition (Wilcoxon signed ranks test, *p *= 0.109).

## Discussions

The confirmation of re-adaptation to the robotic ankle exoskeleton was essential before performing soleus H-reflex tests. Our previous studies [9,14] have shown that subjects reached steady state of powered walking much faster at the second training session (~6 minutes) than the first session (~25 minutes). For this study, 15 minutes of re-familiarization period in the third session was sufficient to ensure the adaptation. In another published study, we documented the results when using catch trials (i.e., turning off the exoskeleton assistance unexpectedly) [57] to assess the presence of negative aftereffects, a benchmark of motor adaptation [58].

Our findings do not support the hypothesis that the normalized amplitude of soleus H-reflex is reduced when training with a robotic ankle exoskeleton under soleus proportional myoelectric control. With short term training, our subjects reduced soleus background EMG by ~35% and had less concomitant reductions in H-reflex amplitude by ~20% during steady-state powered walking. As a result, subjects demonstrated slightly higher H-reflex amplitude relative to their background muscle activity compared to unpowered walking.

The amplitude of the soleus H-reflex depends on presynaptic modulation of Ia afferents (e.g., increased presynaptic inhibition) as well as overall excitability of the motoneuron pool (e.g., a decrease in the voluntary drive of soleus muscle). The unaltered H-reflex modulation in this study indicates that stretch reflex inhibition (i.e., increased presynaptic inhibition of Ia afferents) is likely not one of the mechanisms for reducing soleus EMG when adapting to robotic assistance with short term training. Instead, our results suggest that mechanisms for this short-term adaptation to the robotic assistance could be decreased excitability of the soleus motoneuron pool, resulting from increased inhibition of the motor neurons or a reduction in supra-spinal drive [59].

Adaptation to the robotic exoskeleton assistance during walking may occur in two phases, a quick adaptation that occurs in the first few hours or days and a much longer adaptation that continues for weeks [60-62]. The two adaptation phases may have been reflected by the difference between our current study results on newly trained subjects and the pilot study on a long-term trained subject [51]. When initially walking with the robotic ankle exoskeleton, subjects' gait patterns were greatly disturbed by the additional ankle mechanical torque provided [14]. Decreased motor output of soleus motor neurons due to increased post-synaptic inhibition or a reduction in supra-spinal excitation [63] would be strategies to quickly reduce significant amount of soleus EMG without altering the excitability of reflex pathway. With longer term training, modulation of spinal reflex pathways by supra-spinal centers (i.e., increased pre-synaptic inhibition of Ia afferents) could contribute to soleus EMG reduction without need for constant supraspinal inhibition. The different sensorimotor calibration after long term training may result from repeated motor adaptation to the robotic assistance [61].

During the initial learning of a motor task, increased attention may also enhance the reflex responses. Previous studies have shown greater H-reflex responses during the initial training on a novel locomotion task such as obstacle avoidance during walking [64] and backward walking [41]. In our study, the subjects had trained with the robotic-assisted walking for two thirty-minute sessions and had a 15-minute period of practice with powered walking by the time of H-reflex testing. From subjects' comments after data collection, it seemed that a certain amount of attention or concentration was necessary to walk smoothly with the augmented mechanical plantar flexor torque provided by the exoskeleton at the third session. This may have contributed to the enhanced H-reflex amplitude relative to the background EMG in the powered walking in our study.

## Conclusions

Our findings suggest that the nervous system does not inhibit the soleus H-reflex in response to short-term adaption to exoskeleton assistance as a mechanism for reducing soleus muscle recruitment. Likely mechanisms for the decrease in soleus EMG include spinal or supraspinal post-synaptic inhibition of the soleus motor neurons. Previous results that found H-reflex inhibition in a subject with long term exoskeleton training experience [51] suggest that the neural mechanisms involved in the adaptation to the exoskeleton may change with extended practice. It is unknown how much time or how many repetitions are needed to transition from adapted motor patterns (i.e., motor adaptation) to well learned motor behaviors (i.e., motor learning) [58]. Results from our previous studies suggest that it is faster to achieve steady state performance biomechanically than neurologically [9,14]. Future studies should examine other potential neural mechanisms both in short-term and long-term adaptation to the exoskeleton as considerable evidence suggests that robotic exoskeletons and orthoses have strong potential for improving mobility in patients with neurological impairments [10-13].

## Competing interests

The authors declare that they have no competing interests.

## Authors' contributions

PCK recruited subjects, managed data collections, completed data analysis and drafted the manuscript. CLL developed a custom-written program to control the timing of electrical stimuli, assisted with data analysis and helped edit the manuscript. DPF conceived of the study, provided guidance on experimental design, and helped draft and edit the manuscript. All authors read and approved the final manuscript.
